# 伊马替尼治疗初发慢性髓性白血病慢性期患者获得分子学反应的临床预测模型

**DOI:** 10.3760/cma.j.issn.0253-2727.2023.02.004

**Published:** 2023-02

**Authors:** 子郁 李, 梦雨 张, 小帅 张, 倩 江

**Affiliations:** 北京大学人民医院、北京大学血液病研究所、国家血液系统疾病临床医学研究中心，北京 100044 Peking University People's Hospital, Peking University Institute of Hematology, National Clinical Research Center for Hematologic Disease, Beijing Key Laboratory of Hematopoietic Stem Cell Transplantation, Beijing 100044, China

**Keywords:** 白血病，髓样，慢性, 伊马替尼, 分子学反应, 临床预测模型, Leukemia, myeloid, chronic, Imatinib, Molecular response, Predictive system

## Abstract

**目的:**

建立一线伊马替尼治疗的初发慢性髓性白血病慢性期（CML-CP）患者获得分子学反应的临床预测模型。

**方法:**

收集2006年1月至2022年3月在北京大学人民医院接受伊马替尼一线治疗的成人CML-CP连续病例，将患者按2∶1比例随机分为训练集和验证集。在训练集中，采用竞争风险模型探索主要分子学反应（MMR）及分子学反应4（MR4）的独立影响因素，建立临床预测模型，在验证集中完成内部验证。采用时间依赖性受试者工作特征曲线下面积（AUROC）评价预测模型区分度。

**结果:**

共纳入1 364例患者，训练集909例，验证集455例。在训练集中，多因素分析显示男性、ELTS评分中/高危、初诊时高WBC、低HGB水平与较低的MMR与MR4累积获得率显著相关。根据回归系数建立MMR临床预测模型：男性、ELTS评分中危及低HGB水平（<110 g/L）赋1分，ELTS评分高危、初诊时高WBC（≥130×10^9^/L）赋2分；建立MR4临床预测模型：男性赋1分，ELTS评分中危与低HGB水平各赋2分，初诊时高WBC（≥120×10^9^/L）赋3分，ELTS评分高危赋4分。根据上述预测模型，将患者分为MMR与MR4低、中、高危组，不同危险组累积MMR、MR4获得率差异均具有统计学意义（*P*值均<0.001）。在训练集及验证集中，MMR和MR4预测模型的时间依赖性AUROC范围分别为0.70～0.84和0.64～0.81。

**结论:**

通过联合性别、初诊时WBC、HGB水平和ELTS评分建立了一线伊马替尼治疗的CML-CP患者获得MMR及MR4的临床预测模型，该模型区分度、精确度良好，可帮助指导一线治疗药物的选择。

## Abstract (en)

**Objective:**

To develop a scoring system to predict molecular responses in patients with chronic myeloid leukemia in the chronic phase（CML-CP）receiving initial imatinib therapy.

**Methods:**

Data from consecutive adults with newly diagnosed CML-CP treated by initial imatinib was interrogated and subjects were distributed randomly into training and validation cohort, in a ratio of 2∶1. Fine-gray models were applied in the training cohort to identify co-variates of predictive value for major molecular response（MMR）and MR4. A predictive system was built using significant co-variates. The predictive system was then tested in the validation cohort and the area under the receiver-operator characteristic curve（AUROC）was used to estimate accuracy of the predictive system.

**Results:**

1 364 CML-CP subjects receiving initial imatinib were included in this study. Subjects were distributed randomly into training cohort（*n*＝909）and validation cohort（*n*＝455）. In the training cohort, the male gender, European Treatment and Outcome Study for CML（EUTOS）Long-Term Survival（ELTS）intermediate-risk, ELTS high-risk, high WBC（≥130×10^9^/L or 120×10^9^/L, MMR or MR4）and low HGB（<110 g/L）at diagnosis were significantly related with poor molecular responses and were given points based on their regression coefficients. For MMR, male gender, ELTS intermediate-risk and low HGB（<110 g/L）were given 1 point; ELTS high-risk and high WBC（≥130×10^9^/L）, 2 points. For MR4, male gender was given 1 point; ELTS intermediate-risk and low HGB（<110 g/L）were given 2 points; high WBC（≥120×10^9^/L）, 3 points; ELTS high-risk, 4 points. We divided all subjects into 3 risk subgroups according to the predictive system above. Cumulative incidence of achieving MMR and MR4 in 3 risk subgroups was significantly different in both training and validation cohort（all *P* values <0.001）. In the training and validation cohorts, the time-dependent AUROC ranges of MMR and MR4 predictive systems were 0.70-0.84 and 0.64-0.81, respectively.

**Conclusion:**

A scoring system combining gender, WBC, HGB level and ELTS risk was built to predict MMR and MR4 in CML-CP patients receiving initial imatinib therapy. This system had good discrimination and accuracy, which could help phsicians optimize the selsction of initial TKI-therapy.

自酪氨酸激酶抑制剂（TKI）问世以来，慢性髓性白血病（CML）患者的预期寿命接近正常人[1]，CML的治疗目标也随之发生改变[2]–[3]。越来越多的患者可获得持续且稳定的主要分子学反应（MMR）甚至深度分子学反应（DMR）。伊马替尼是上市时间最长和使用人数最多的TKI[4]。研究显示，获得稳定的MMR意味着较低的疾病进展率[2],[5]，获得分子学反应4（MR4）则是追求无治疗缓解（TFR）的前提[2],[6]。因此，精确预测CML慢性期（CP）患者在一线伊马替尼治疗下获得MMR或MR4的可能性对一线药物的选择有一定的帮助。既往研究发现，性别、血红蛋白水平、WBC、BCR-ABL转录本类型及Sokal、Hasford、EUTOS、ELTS评分等因素与CML-CP患者的分子学反应获得率显著相关[7]–[13]。但目前仍缺乏明确的预后积分系统用于预测一线伊马替尼治疗下CML-CP患者的分子学反应获得率。因此，我们系统性回顾并收集在我院就诊的1 364例接受一线伊马替尼治疗的CML-CP患者的初诊及治疗随访信息，以开发并验证预测MMR与MR4获得率的积分系统。

## 病例与方法

1. 病例：回顾性收集从2006年1月至2022年3月在北京大学人民医院就诊的≥18岁且携带e14a2和（或）e13a2 BCR-ABL转录本、一线使用伊马替尼治疗的初诊CML-CP患者资料，包括人口学与临床变量：性别、年龄、全血细胞计数、脾脏大小、合并症、各线TKI种类、剂量与换药原因及服药后血液学、细胞遗传学、分子学监测随访结果等。所有资料均为初诊未服用任何降细胞药物时的数据。

2. 诊断标准、治疗、监测、治疗反应与结局定义：患者诊断、分期、一线治疗选择及监测均参照欧洲白血病网（ELN）指南[2]–[3]。

治疗反应定义：①完全血液学反应（CHR）：WBC<10×10^9^/L，PLT<450×10^9^/L，外周血无原始细胞或早幼粒细胞，中幼粒细胞+晚幼粒细胞<5％，嗜碱性粒细胞比例<5％，无髓外白血病表现，上述条件应至少持续4周；②完全细胞遗传学反应（CCyR）：至少在20个分裂中期的骨髓细胞中未见Ph染色体；③MMR：BCR-ABL^IS^≤0.1％；④MR4：BCR-ABL^IS^≤0.01％。至少连续两次检测确认BCR-ABL水平才能被定义为稳定的分子学反应。确认获得MMR或MR4后，BCR-ABL^IS^>0.1％被定义为丧失MMR，BCR-ABL^IS^>0.01％为丧失MR4。

结局定义：①无治疗失败生存（FFS）期：从开始TKI治疗至治疗失败或末次随访的时间；②无疾病进展生存（PFS）期：从开始TKI治疗至进展到加速期/急变期、死亡或末次随访的时间；③总生存（OS）期：从开始TKI治疗至死亡或末次随访的时间。所有结局观察均删失至造血干细胞移植。

3. 随访：采用门诊或电话联系的方式进行随访，随访截止时间为2022年3月。

4. 统计学处理：患者基线信息包括人口学资料与临床特征采用描述性统计分析，分类变量用频数和频率进行描述，连续变量用中位数（范围）或中位数（IQR）进行描述。针对连续变量采用时间依赖性受试者工作特征曲线（ROC）确定其预测分子学反应累积获得率的最佳截断值。所有患者按2∶1比例随机分至训练集和验证集。在训练集中，采用竞争风险模型探索MMR、MR4累积获得率的独立影响因素，单因素分析*P*<0.2的变量纳入多因素分析。采用共线性诊断检验各变量间共线性情况。依据模型中各变量的回归系数赋分建立预测模型[14]。对于累积治疗反应获得率采用竞争风险模型分析，以移植、非CML相关死亡、停药、换药作为竞争风险事件，并应用Fine-Gray检验进行组间比较[15]。使用时间依赖性受试者工作特征曲线下面积（AUROC）评价预测模型区分度[16]–[17]。*P*<0.05为差异有统计学意义。分别采用SPSS 22.0及R 4.0.2软件进行统计分析、绘图。

## 结果

1. 患者特征：共收集到1 620例接受一线伊马替尼治疗的CML-CP连续性病例资料，排除诊断至开始治疗时间>6个月16例、关键临床信息缺失122例、不规律随访或失访92例、非e13a2或e14a2转录本类型26例；最终本研究纳入具有完整临床信息和随访资料的1 364例接受一线伊马替尼治疗的CML-CP患者。中位年龄41（18～83）岁，男性840例（61.6％）。ELTS评分低危926例（67.9％）、中危331例（24.3％）、高危107例（7.8％）。初诊时伴高危附加染色体异常33例（2.8％），伴合并症509例（37.3％）（表1）。

**表1:** 1 364例接受一线伊马替尼治疗的慢性髓性白血病慢性期患者的基线特征

特征	患者总体（1 364例）	训练集（909例）	验证集（455例）	统计量	*P*值
年龄［岁，*M*（范围）］	41（18～83）	41（18～83）	41（18～83）	−0.469	0.639
男性［例（％）］	840（61.6）	567（62.4）	273（60.0）	0.724	0.395
ELTS评分［例（％）］				0.406	0.816
低危	926（67.9）	612（67.3）	314（69.0）		
中危	331（24.3）	224（24.6）	107（23.5）		
高危	107（7.8）	73（8.0）	34（7.5）		
WBC［×10^9^/L，*M*（范围）］	114.5（2.3～785.6）	109.2（2.3～785.6）	122.4（4.5～698.8）	−0.543	0.587
HGB［g/L，*M*（范围）］	116（28～340）	117（28～340）	115（49～183）	−1.899	0.058
PLT［×10^9^/L，*M*（范围）］	406（36～3 707）	403（36～3 707）	414（90～2 361）	−0.896	0.370
外周血原始细胞［％，*M*（范围）］	1（0～14）	1（0～14）	1（0～14）	−0.082	0.935
外周血嗜碱性粒细胞［％，*M*（范围）］	4（0～19）	5（0～19）	4（0～19）	−1.811	0.070
伴高危ACA［例（％）］	33（2.8）	24（3.0）	9（2.2）	0.800	0.371
伴合并症［例（％）］	509（37.3）	331（36.4）	178（39.1）	0.950	0.330
随访时间［月，*M*（范围）］	50（3～193）	50（3～193）	49（6～182）	−0.168	0.867

**注** ACA：附加染色体异常

截至末次随访，330例（24.2％）因治疗失败（240例）、不耐受（47例）或本人意愿（43例）转换为二代TKI（尼洛替尼206例，达沙替尼109例，普纳替尼9例，奥雷巴替尼6例）；其余1 034例（75.8％）仍接受伊马替尼治疗。

中位TKI治疗时间为53（IQR 32～82）个月，累积获得MMR 837例（61.4％）、MR4 528例（38.7％）。截至末次随访，29例丧失MMR，46例丧失MR4；344例治疗失败，125例疾病进展，54例死亡（包括49例CML相关死亡，5例其他原因死亡）。7年MMR和MR4累积获得率分别为71.4％（68.4％～74.4％）和55.1％（52.3％～57.9％），7年FFS率、PFS率和OS率分别为72.7％（69.6％～75.8％）、89.1％（86.8％～91.4％）和94.7％（93.6％～95.8％）。

1 364例患者按2∶1比例随机分至训练集（909例）和验证集（455例），两组的基线特征差异均无统计学意义（表1）。

2. 建立MMR临床预测模型：在训练集中，多因素分析显示男性、ELTS评分中/高危、WBC ≥ 130×10^9^/L以及HGB<110 g/L和较低的MMR累积获得率相关（表2），共线性诊断提示变量间无显著共线性［方差膨胀因子（Variance Inflation Factors, VIF）1.0～1.6］。根据各因素回归系数赋分建立MMR预测模型：女性、ELTS评分低危、WBC<130×10^9^/L和HGB≥110 g/L赋0分；男性、ELTS评分中危和HGB<110 g/L赋1分；ELTS评分高危和WBC ≥ 130×10^9^/L赋2分。根据上述赋分规则，将909例患者分为0分（141例，15.5％）、1分（288例，31.7％）、2分（79例，8.7％）、3分（123例，13.5％）、4分（138例，15.2％）、5分（106例，11.7％）和6分（34例，3.7％）组。由于0～1分组、2～4分组、5～6分组内各亚组MMR累积获得率差异无统计学意义，最终将909例患者分为三组：低危组（总分0～1分，429例，47.2％）、中危组（总分2～4分，340例，37.4％）和高危组（总分5～6分，140例，15.4％）。各组7年的MMR累积获得率分别为86.3％（82.4％～90.2％）、62.1％（56.2％～68.0％）和33.8％（25.3％～43.3％）（图1A，*P*<0.001）；以低危组为参考，中、高危组获得MMR的风险比（*HR*）分别为0.411（95％ *CI* 0.346～0.487，*P*<0.001）和0.178（95％ *CI* 0.129～0.248，*P*<0.001）（总体*P*值< 0.001）。

**表2:** 接受一线伊马替尼治疗的慢性髓性白血病慢性期患者获得主要分子学反应（MMR）和分子学反应4（MR4）的多因素分析结果（训练集）

因素	MMR				MR4			
	回归系数	*HR* (95％*CI*)	*P*值	赋分	回归系数	*HR* (95％*CI*)	*P*值	赋分
男性	−0.350	0.705（0.595～0.835）	<0.001	1	−0.282	0.754（0.605～0.942）	0.013	1
ELTS评分								
低危（参考）		1				1		
中危	−0.464	0.625（0.498～0.785）	<0.001	1	−0.509	0.601（0.442～0.817）	0.001	2
高危	−0.743	0.475（0.312～0.723）	<0.001	2	−1.212	0.298（0.146～0.606）	<0.001	4
WBC≥130 ×10^9^/L	−0.538	0.593（0.492～0.716）	<0.001	2	−0.747	0.474（0.369～0.607）	<0.001	3
HGB<110 g/L	−0.490	0.613（0.497～0.756）	<0.001	1	−0.496	0.609（0.458～0.808）	<0.001	2

**图1:**
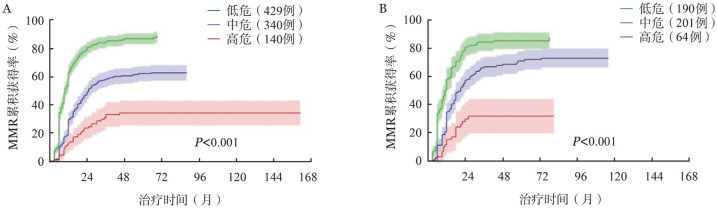
接受一线伊马替尼治疗的慢性髓性白血病慢性期患者训练集（A）和验证集（B）的主要分子学反应（MMR）累积获得率

在验证集中，455例患者根据上述MMR临床预测模型分为低危组（190例，41.8％）、中危组（201例，44.2％）和高危组（64例，14.1％）。各组7年的MMR累积获得率分别为87.8％（80.4％～91.2％）、71.6％（64.7％～78.5％）和31.3％（19.2％～43.4％）（图1B，*P*<0.001）。以低危组为参考，中、高危组获得MMR的*HR*值分别为0.469（95％ *CI* 0.378～0.583，*P*<0.001）和0.256（95％ *CI* 0.170～0.385，*P*<0.001）（总体*P*值<0.001）。

3. 建立MR4临床预测模型：在训练集中，多因素分析显示男性、ELTS评分中/高危、WBC ≥ 120×10^9^/L以及HGB<110 g/L和较低的MR4累积获得率相关（表2）；共线性诊断提示变量间无显著共线性（VIF 1.0～1.6）。根据各因素的回归系数赋分建立MR4预测模型：女性、ELTS评分低危、WBC<120×10^9^/L和HGB ≥ 110 g/L赋0分；男性赋1分；ELTS评分中危和HGB<110 g/L赋2分；WBC ≥ 120×10^9^/L赋3分；ELTS评分高危赋4分。根据上述赋分规则，909例患者被分为三组：低危组（总分0～2分，415例，45.7％）、中危组（总分3～7分，352例，38.7％）和高危组（总分8～10分，142例，15.6％），7年的MR4累积获得率分别为79.2％（74.1％～84.3％）、41.8％（35.8％～47.8％）和19.1％（10.3％～27.9％）（图2A，*P*<0.001）。以低危组为参考，中、高危组获得MR4的*HR*值分别为0.334（95％ *CI* 0.267～0.419，*P*<0.001）和0.142（95％ *CI* 0.090～0.223，*P*<0.001）（总体*P*值<0.001）。

**图2:**
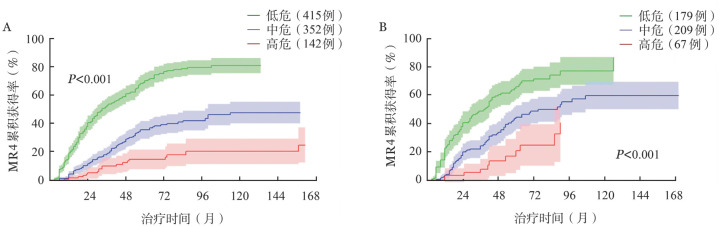
接受一线伊马替尼治疗的慢性髓性白血病慢性期患者训练集（A）和验证集（B）的分子学反应4（MR4）累积获得率

在验证集中，455例患者根据上述MR4临床预测模型分为低危组（179例，39.3％）、中危组（209例，45.9％）和高危组（67例，14.7％）。7年的MR4累积获得率分别为74.4％（65.4％～83.4％）、50.1％（41.4％～58.8％）和24.7％（10.1％～39.3％）（图2B，*P*<0.001）。以低危组为参考，中、高危组获得MR4的*HR*值分别为0.466（95％ *CI* 0.348～0.624；*P*<0.001）和0.191（95％ *CI* 0.107～0.341；*P*<0.001）（总体*P*值<0.001）。

4. 临床预测模型的评价：采用时间依赖性ROC曲线评价MMR和MR4临床预测模型区分度，均显示出较高的灵敏度和特异度。在训练集中，MMR临床预测模型的AUROC在1年、3年和5年时分别为0.77（0.73～0.81）、0.70（0.65～0.75）和0.80（0.76～0.84），MR4临床预测模型的AUROC在1年、3年和5年时分别为0.75（0.71～0.79）、0.71（0.66～0.76）和0.79（0.75～0.83）。在验证集中，MMR临床预测模型的AUROC在1年、3年和5年时分别为0.74（0.70～0.78）、0.79（0.75～0.83）和0.84（0.79～0.89），MR4临床预测模型的AUROC在1年、3年和5年时分别为0.81（0.73～0.89）、0.73（0.67～0.79）和0.64（0.60～0.68）。

## 讨论

本研究通过联合性别、初诊时WBC、HGB浓度和ELTS评分，建立了针对MMR与MR4的简易临床预测模型，模型中涉及的参数信息易于获得，评分易于计算，有助于临床医师预测初诊CML-CP患者采用一线伊马替尼治疗后获得MMR及MR4的可能性。

既往研究报道，初诊时高WBC、低HGB、外周血嗜碱性粒细胞比例高、ELTS/Sokal评分中高危、某些类型转录本以及未达到早期分子学反应与一线伊马替尼治疗CML-CP患者更低的分子学反应累积获得率相关。Ko等[9]研究154例一线伊马替尼治疗的CML-CP患者显示，相较于Sokal评分，初诊时中度贫血（HGB<100 g/L）能更好地预测MR4.5，联合初诊时HGB浓度与3个月时早期分子学反应（3M-EMR）可以更好地预测MR4.5。一项来自意大利的研究纳入559例一线伊马替尼治疗的CML-CP患者，研究显示具有e14a2转录本的患者MR4的中位获得时间显著短于e13a2转录本患者[13]。我中心的一项研究显示，3个月时高BCR-ABL^IS^基因水平（≥10％），初诊时高WBC（>150×10^9^/L）、低HGB（<120 g/L）以及男性与较低的MR4.5累积获得率显著相关，Sokal评分不是MR4.5的独立影响因素[8]。并且，既往研究发现，对于接受一线伊马替尼治疗的CML-CP患者，ELTS评分相较于Sokal评分能够更好地预测患者的MMR、MR4以及MR4.5[11]。本研究发现，男性、ELTS中/高危、初诊时高WBC以及低HGB浓度与较低的MMR和MR4获得率显著相关，与以往研究相似。本研究通过联合上述因素建立了简易可行的临床预测模型，相较于既往研究所报道的独立影响因素，AUROC较高，可重复性高。此外，本研究中预测模型纳入ELTS积分而不是Sokal积分，是因为已有研究证明ELTS积分对于分子学反应预测的优越性[11]。

本研究有以下局限性：①为单中心、回顾性研究；②本研究中，未区分e14a2与e13a2转录本，这可能是影响治疗反应的一个因素；③本研究未纳入患者服药依从性的监测，后者可能会影响患者的治疗反应获得率。

总之，我们联合性别、初诊时WBC、HGB浓度和ELTS评分建立了一个具有良好区分度、精确度、用于预测初诊CML-CP患者接受一线伊马替尼治疗获得MMR和MR4的简易临床评分系统。该模型能帮助医师在初诊时识别高危患者，予以更多的关注和管理，制定切合实际的治疗目标，并指导一线治疗药物的选择，进一步改善患者的整体治疗结局，为个体化精准管理和治疗提供重要依据。
